# Effects of Exogenous Application of Protocatechuic Acid and Vanillic Acid to Chlorophylls, Phenolics and Antioxidant Enzymes of Rice (*Oryza sativa* L.) in Submergence

**DOI:** 10.3390/molecules23030620

**Published:** 2018-03-09

**Authors:** Tran Dang Xuan, Do Tan Khang

**Affiliations:** 1 Graduate School of International Development and Cooperation, Hiroshima University, Higashi-Hiroshima City 739-8529, Japan; 2 Biotechnology Research and Development Institute, Can Tho University, Can Tho City 902070, Vietnam; dtkhang@ctu.edu.vn

**Keywords:** antioxidant enzymes, exogenous application, root treatment, protocatechuic acid, submergence tolerance, rice, vanillic acid, mixture

## Abstract

In this study, effects from application of protocatechuic acid (PA) and vanillic acid (VA) and their mixture on the submergence tolerance of rice were examined. The treatment of 0.01 mM PA and VA did not show significant increase of rice growth as compared to the controls. However, at higher concentrations (0.1–1.0 mM), rice shoot was elevated in submergence by 20.8–22.4%. The survival percentage of rice seedlings at any dose of PA, VA and their mixture was significantly higher than the controls. In general, the mixture of PA and VA was more active to promote shoot elongation and survival in submergence than sole treatment of either PA or VA. The amount of chlorophyll *b* by PA was significantly increased, while no change in chlorophyll *a* content was observed. VA remarkably reduced malondialdehyde quantity at three days of submergence, while no significant difference among treatment was observed in PA, the mixture, and respective controls. The two phenolic acids promoted contents of phenolics and flavonoids in rice leaves and roots, however the quantities of endogenous PA and VA in rice were not markedly differed after PA and VA treated on roots of rice seedlings. The ascorbate peroxidase and superoxide dismutase activities were enhanced, while the expression of genes encoding antioxidant enzymes was favored. VA increased the expression level of ascorbate peroxidase genes in higher levels than PA and their mixture, while no significant difference was observed in the other genes including superoxide dismutase, catalase, glutathione reductase, and peroxidase. Findings of this study showed that PA and VA increased the submergence tolerance of rice by promoting the photosynthetic and anti-oxidative processes in rice seedlings. The treatment of PA and VA mixture on seedling roots was potent to promote the submergence tolerance in rice.

## 1. Introduction

Submergence substantially causes major limitations on productivity and viability of agricultural crops worldwide. Among important crops, rice (*Oryza sativa* L.) is mostly affected by submergence stress since many rice landraces are primarily cultivated in lowland and flood-prone areas [1]. There are annually more than 20 million hectares of paddy fields in the world exposed to flood or submergence [2]. The Mekong and Red River deltas of Vietnam, the Ayeyerwaddy delta of Myanmar and the Ganges-Brahmaputra delta of Bangladesh, the central rice production areas worldwide, have been faced with submergence [1,3].

In recent years, many submergence-tolerant rice varieties have been generated using both conventional and DNA marker-assisted selection approaches. The *SUB1* QTL (quantitative locus trait) [4], which included three ethylene response factor-like genes (*SUB1A*, *SUB1B*, and *SUB1C*) [4,5], and controlled by the *SUB1* gene determining the submergence tolerance, was identified. In addition, the *SNORKEL1* and *SNORKEL2* genes [6] which involved in the rapid elongation response of deep water rice, respectively, were detected. Success in using the *SUB1* gene has provided the development of many submergence tolerance varieties [2]. However, the fact that many varieties make limited recovery after submergence exposure is one of the practical problems of those methods. Specifically, limited recovery may detrimentally affect physiological development and reproduction with significant reductions in the total productivity [7]. In addition, the moderately submergence-tolerant varieties are normally low in both yield and quality [8]. Therefore, improving the submergence tolerance of the current rice varieties by enhancing their physiological processes using exogenous regulatory factors during submergence is one of the promising solutions [9].

Ecologically, rice can survive under water for a short period due to adaptive physiological mechanisms including enzymatic and non-enzymatic factors [10,11]. Regarding the former factor, an antioxidant enzyme system has been discovered in plants subjected to biotic and abiotic stresses producing numerous varieties of activated oxygen species consisting of superoxide, singlet oxygen, hydroxyl radicals, and hydrogen peroxide [12,13]. In fact, superoxide dismutase (SOD) is initially activated to convert superoxide to oxygen and hydrogen peroxide. The three major enzymes, namely catalase (CAT), glutathione peroxidase (GPx), and ascorbate peroxidase (APX) subsequently cleavage hydrogen peroxide, a dangerous oxygen-containing free radical, into water molecules [14,15,16,17]. Damanik et al. [16] highlighted the increase of SOD, CAT, APX and glutathione reductase (GR) activities in Malaysian rice cultivars after eight days of complete submergence, and supposed that if these antioxidant enzyme activities were promoted during excess water stress, the level of tolerance would be increased. In terms of the non-enzymatic mechanism in submergence responses, phenolics have been reported to have strong scavenging capacity of free radicals relating to oxidative stress [18]. Particularly, a marked increase in total phenolic content was recorded in plants during exposure to UV light [19,20], saline stress [21,22], drought stress [23], and flooding condition [24].

It is estimated that over 20 million ha in rainfed lowland areas are detrimentally influenced by flood annually [2]. These areas are subjected to either frequent flash flood or submergence, including longer-term flooding (20–50 cm) (partial/stagnant, semi-deep), deep water >100 cm, and very deep water (up to 3–4 m) (in floating rice areas). Rice productivity has been adversely reduced (averaging about 1.5 tons/ha) because of the lack of rice cultivars tolerant to submergence stress [2]. The flooding can be classified into: flooding during germination (anaerobic germination), a problem when direct seeding is practiced and affected by heavy rain; flash flood (submergence), where rice plants are completely submerged for up to two weeks, thus submergence tolerance is required for this condition; stagnant flooding, where the water depth is up to 50 cm, and flooding occurs more than two weeks to several months, hence rice varieties tolerant of stagnant flooding are required; and deeper stagnant flooding, where the water depth is from >50 cm to 1 m or more. That needs rice cultivars with tall plant height or rapid internode elongation [2]. However, the anaerobic flooding during germination and flash flood caused detrimental reduction on rice growth and yields, as rice cultivars being cultivated are often non-submergence tolerance. Countermeasures to lower the harmful affect attributed to anaerobic flooding and submergence are needed.

There have been several attempts to increase the submergence tolerance of rice. For example, post-flood N application either as broadcasting or spray markedly promoted survival, photosynthesis, anti-oxidant activity, and rice yield, while pre- and post-submergence Si spray detrimentally affected elongation, lodging, leaf senescence, and chlorosis [25]. Ethylene-air treatment stimulated internode elongation, and thus stimulated growth in submerged floating rice plants [26]. Nutrient application at the seedling stage [27], and post-submergence N and basal P application increased plant survival and productivity of rice [25]. However, either pre- or post-application of nutrients may severely affect rice yield and quality and require heavy labor force in fields. 

In our previous study, it was reported that the quantities of protocatechuic acid (PA) and vanillic acid (VA) in rice seedlings were significantly increased (6.50 and 2.89 folds, as compared to the non-treated rice, respectively) when rice seeds were treated in anaerobic flood during germination [24]. However, the responses of the two compounds to the water depth (5 and 10 cm) differed. This study was therefore conducted to examine the exogenous applications on rice roots of PA, VA, and their mixture to examine the efficacy on the elevation to rice growth in submergence. The influences on chlorophyll contents, lipid peroxidation, antioxidant enzyme activities and gene expression were also investigated. 

## 2. Results

### 2.1. Effects of Exogenous Application of PA and VA on Rice Growth

Table 1 shows the effects from treatments (0.01, 0.1, and 1.0 mM doses) of PA, VA and their mixtures on shoot height of rice. The elevation of shoot height was the most important in submergence tolerance, thus only the criterion of rice emergence was measured. At the lowest dose (0.01 mM), both PA and VA did not significantly elevate shoot height of rice, but their mixture promoted rice growth by 15.7% in submergence, as compared to the respective controls (Table 1). The shoot height was remarkably promoted by 0.1–1.0 mM doses by single treatments of either PA or VA, and their mixture, however the levels of elevation were not significantly different among applied doses and compounds. Findings in Table 1 revealed that the mixtures of PA and VA were more effective to elevate rice growth in submergence than either sole PA or VA treatment. The dose 0.1 mM was therefore selected to examine the changes in chemical components, antioxidant enzymes and gene expression by PA and VA application.

Results in Table 1 showed that the treatments of PA and VA at any dose significantly increased the survival percentage of rice in submergence. At 1.00 mM of PA, and mixture at 0.10–1.00 mM of the mixture, no injury on rice survival was observed. Both PA and VA showed similar tolerance level at 0.10 mM, which was lower than their mixture. At 0.01 mM, both PA and VA exhibited comparable tolerance strength that was remarkably higher than the control, but was lower than their mixture. The mixture of PA and VA at any dose exhibited higher survival percentage than either PA or VA (Table 1). Regarding the evaluation by survival scale, application of VA and PA at 0.01 mM did not show different survival scale compared to the control (Table 1). However, at 0.10–1.0 mM, both VA and PA increase the survival of rice seedling, and PA showed greater survival at the maximal dose (1.0 mM) than VA. Combination of both PA and VA showed greater survival strength than the single application of either PA or VA (Table 1).

### 2.2. Effects of Exogenous Application of PA and VA Photosynthetic Pigments and Lipid Peroxidation

**Figure 1:**
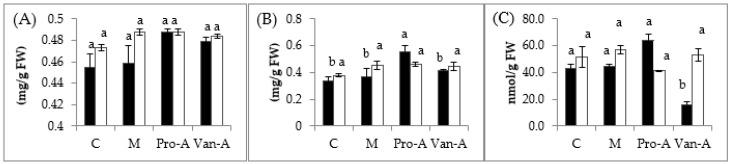
Changes of: chlorophyll *a* (**A**); chlorophyll *b* (**B**); and MDA (**C**) contents of rice after pre-treatment with PA and VA. Black column (three days after submergence) and white column (six days after submergence); C: Control, M: Mixture, Pro-A: Protocatechuic acid, Van-A: Vanillic acid; Means with different small letters in the same column color are significantly different (*p* < 0.05); FW: Fresh weight; MDA: malondialdehyde.

Figure 1 shows the content of chlorophyll *a* and *b* in rice seedlings under submergence with phenolic acid pre-treatment. After 24 h soaking with 0.1 mM PA, VA, and the mixture, the chlorophyll *a* in rice leaves was maintained at a steady level at either three days or six days after pre-treatment. However, the content of chlorophyll *b* was 40% higher than that of the control (*p* < 0.05) after rice seedlings treated with PA for three days. After three days, VA caused a significant reduction of >50% in the malondialdehyde (MDA) content (*p* < 0.05), while the content of each of phenolic acid treatment after six days was still relatively comparable to that of the control (Figure 1).

### 2.3. Effect of Exogenous Application of PA and VA on Total Contents of Phenolics and Flavonoids, and Endogenous PA and VA 

**Figure 2:**
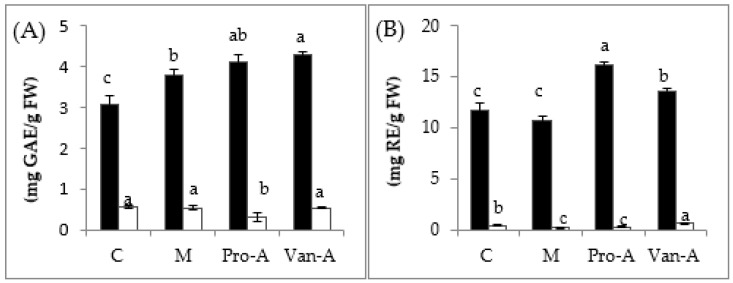

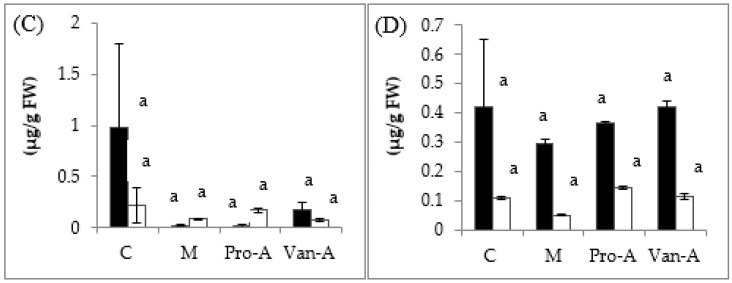
Variation of: total phenolic (**A**); total flavonoid (**B**); protocatechuic acid (**C**); and vanillic acid (**D**) contents in rice after pre-treatment with PA and VA. Black column (leaves) and white column (roots). Means with different small letters in the same column color are significantly different (*p* < 0.05); FW: Fresh weight, GAE: Gallic acid equivalent, RE: Rutin equivalent.

The total phenolic and flavonoid contents of rice seedlings considerably were changed during submergence after pre-soaking in exogenous PA and VA (Figure 2). All treatments significantly improved the total phenolic contents in leaves (*p* < 0.05). In contrast, the tremendous reduction of both total phenolic and flavonoid contents was recorded in roots after pre-treatment. Total phenolic contents were significantly increased in rice leaves by PA, VA, and their mixtures. However, total flavonoids were markedly promoted only by sole application of either PA or VA, whereas their mixture was statistically similar to the respective control (Figure 2). Treatments of either PA or VA, or their mixture did not change endogenous contents of PA and VA in rice leaves and roots (Figure 2).

### 2.4. Effect of Exogenous Phenolics on Antioxidant Enzymes

**Figure 3:**
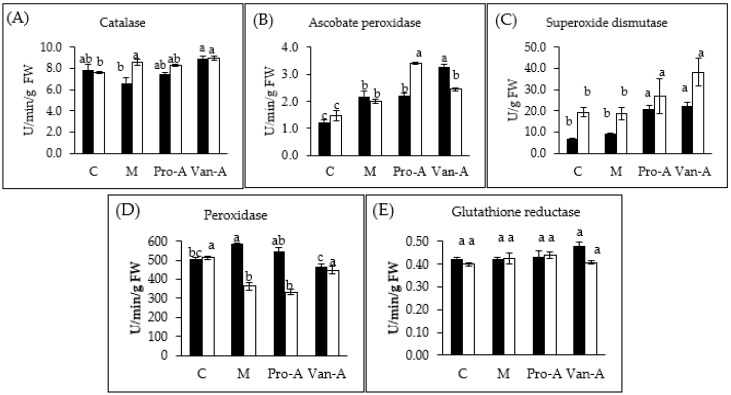
Changes of: catalase (**A**); ascobate peroxidase (**B**); superoxidase (**C**); peroxidase (**D**); and glutathione reductase (**E**) activities in rice after pre-treatment with PA and VA. Black column (three days after submergence) and white column (six days after submergence). Means with different small letters in the same column color are significantly different (*p* < 0.05).

The changes in antioxidant enzyme activities after pre-soaking with phenolic acids are shown in Figure 3. The catalase activity was not affected by the phenolic acid pre-treatments in submergence stress after three days. However, after six days, the catalase activity greatly increased by VA and the mixture pre-treatments (*p* < 0.05). The SOD activity was markedly enhanced with both PA and VA pre-treatment, which was more than three-fold increase compared to the control after three days. All treatments significantly increased the activity of APX after three days and six days of submergence. PA pre-treatment substantially increased the APX activity after three days. However, after six days, the rise of the APX activity was caused by protocatechuic acid pre-treatment. Regarding to the peroxidase (POD) activity, the mixture was more effective than either PA or VA increasing the activity by approximately 14%, compared to the control (*p* < 0.05). No influence of PA, VA, and their mixture on the glutathione reductase (GR) activity was found.

The transcript of antioxidant enzyme genes was slightly enhanced by the phenolic acid treatments compared to the control. Most of the relative expression levels were lower than 1, except APX gene under the VA treatment, which was significantly higher than the PA and the mixture treatments. There was no significant difference in the expression of superoxidase (SOD), catalase (CAT), POD, and GR among three treatments.

### 2.5. Coefficient Correlations among Contents of Total Phenols, Flavonoids, Chlorophylls a and b, Lipid Peroxidation, and Gene Expression of Antioxidant Enzymes

Results in Table 2 showed that the *R^2^* values of total phenolics were significantly proportional to contents of total flavonoids (0.579) and chlorophyll *a* (0.579) at *p* < 0.05, and SOD (0.818) and APX (0.865) at *p* < 0.01, whereas no significance difference with chlorophyll *b* and CAT, POD, and GR was observed. Total flavonoids were markedly proportional to contents of chlorophyll *a* (0.640, *p* < 0.05), and chlorophyll *b* (0.797, *p* < 0.01). Significantly increases were observed in the lipid peroxidation and POD (0.680, *p* < 0.01), chlorophyll *a* and SOD (0.644, *p* < 0.05), SOD and APX (0.721, *p* < 0.05), whereas reverse reduction was observed in CAT and POD (−0.773, *p* < 0.01), and POD and GR (0.702, *p* < 0.05). The contents of chlorophylls *a* and *b* were significantly proportional (0.832, *p* < 0.01) (Table 2).

## 3. Discussion

In our previous study, thirty rice cultivars were treated at 5 and 10 cm anaerobic flooded condition, and it was found that the cultivar Koshihubo was the maximum tolerant [24]. It was found that the number and quantity of phenolic acids were strongly increased during treatment, but they were varied among compounds and depth levels of the anaerobic flooding. In detail, five phenolic acids, PA, chlorogenic acid, VA, benzoic acid, and cinnamic acid, were found in the controls (non-treated plants), of which contents of PA, benzoic acid, and cinnamic acid were significantly increased at 10 cm depth, while quantities of VA and chlorogenic acid exerted remarkable extents at 5 cm depth, but were significantly reduced at 10 cm depth. PA was selected because the accumulation of this compound was higher (6.50 folds), than benzoic acid (1.77 folds) and cinnamic acid (2.91 folds). Similarly, that of VA was higher (2.89 folds) than chlorogenic acid (2.68 folds) [24]. The two compounds were proposed to play a role in the tolerance of rice in anaerobic flooding germination but the responses to the depth of anaerobic flooding (5 and 10 cm) were differed. Hence, PA and VA were used to examine their efficacy on the submergence tolerance in this study.

It was found that the application of PA, VA and their mixtures showed potent stimulation on rice growth in submergence, however the elevated levels were dose-dependent. At 0.01 mM, the mixture showed stronger stimulation on elongation of rice shoot than sole application of either PA or VA. Although no significant difference among 0.01, 0.1 and 1.0 mM doses of the mixture was observed, in general, the mixture at 1.0 mM exerted remarkably higher elevation than the dose 0.1 mM of either PA or VA (Table 1). Findings in Table 1 indicated that the mixture of PA and VA was more active to stimulate rice growth in submergence than individual treatments of either PA or VA. 

Chlorophylls play an extremely crucial role in plant photosynthesis. Numerous previous studies reported that, under submergence stress, the chlorophyll content in leaves massively declined due to high ethylene concentration [28] or carbohydrate accumulation, or light and oxygen insufficiencies [29,30,31,32,33,34]. Wu and Yang [35] reported the significant decrease of chlorophyll *a* and *b* and total contents after a submergence treatment of 14-day old rice seedlings. Moreover, chlorophyll reduction greatly influences on photosynthetic efficiency affecting the normal growth of the plant [9,36]. In this study, the chlorophyll *b* content was considerably alleviated by exogenous protocatechuic acid pre-treatment. A similar study also reported the enhance of chlorophyll content due to phenolic acid (salicylic acid) pre-soaking [36]. Briefly, PA and VA pre-treatments could increase the level of chlorophyll content that would result in the photosynthetic improvement during submergence period. Basically, chlorophyll content is degraded by many environmental factors, e.g., submergence stress. The degradation of chlorophyll is regulated by an enzyme system, and chlorophyllase is one of the most important enzymes [37]. Yang et al. [38] reported that phenolic acids including *o*-hydroxyphenyl acetic, ferulic and *p*-coumaric acids with high concentrations (50 to 200 ppm) significantly stimulated the activities of chlorophyllase *a* and *b* of rice seedlings. This finding proved that there is a close correlation between phenolics and chlorophyll contents in rice. Therefore, the increase of the chlorophyll *b* content might involve in the inhibitory activity of protocatechuic acid on chlorophyllase activity the applied dose was low (0.1 mM), equivalent to about 0.015 ppm. Moreover, the changes of chlorophyll *a* and *b* contents positively correlated to the total endogenous phenolic contents. It could be concluded that the treatments of PA and VA indirectly activated the increase of endogenous phenolics and flavonoids correlated to the non-enzymatic anti-oxidative mechanism of rice under submergence.

Under stress conditions, lipid peroxidation occurs in plant cells and results in severe injuries in plant tissues. MDA, a reactive aldehydic product, is formed by lipid peroxidation during degradation process in biological cells [9,39]. Lu et al. [40] reported that the lower is the MDA content detected in tissues, the lesser is the cell damage that takes place during exposing to stressful conditions. As a result, the level of tolerance was improved [16]. The result in this study highlighted that exogenous VA pre-treatment notably reduced MDA content in rice under submergence at the seedling stage. One of possible reasons for the reduction of lipid peroxidation in rice was the substantial increase of total phenolic and flavonoid contents after being pre-soaked with PA and VA. Basically, among secondary metabolites, phenolics are one of the most important classes of compounds possessing antioxidant activity, especially lipid peroxidation inhibition. The presence of hydroxyl and carbonyl groups (playing a major role in neutralizing free radicals) in an aromatic ring is the most vital characteristic of this group of bioactive compounds [41]. In the case of lipid peroxidation, phenolic compounds lock the lipid alkoxyl radicals, and the more hydroxyl groups a phenolic compound has in the aromatic ring, the more radicals would be trapped [42]. In addition, VA and PA are strong antioxidants abundantly found in plants [43]. Although the central role of exogenous PA and VA in this case was not studied, they were relevant factors in promoting tolerant capacity of rice under submergence.

Apart from phenolics, it is reported that an antioxidant enzyme system—another well-known enzymatic mechanism of plant to overcome environmental stresses—probably involving in the decrease of MDA content in rice seedlings [16,17]. The most effective enzymes consist of SOD, CAT, APX, POD, and GR. Among antioxidant enzymes, SOD is the most abundant in plant because it can be found in the cell wall, cytosol, peroxisome, microsome, apoplast, mitochoria and chloroplast of a cell [13]. In this study, the activity of most antioxidant enzymes (except GR) was significantly increased during submergence after pre-treatment with PA and VA. Apparently, the increase of some antioxidant enzyme activities was proportional to the rise of total phenolic and flavonoid contents. Particularly, the total phenolic content had high correlations to the SOD and APX activities, while total flavonoid content strongly correlated to the SOD activity (Table 2). In fact, the activities of those antioxidant enzymes and total phenolic and flavonoid contents increased during abiotic and biotic stresses, as previously reported [16,19,20,21,22,23,24]. For example, SOD, CAT, APX and GR activities of rice seedlings were greatly stimulated after submergence [16,17]. In comparison with previous works, under hypoxia stress, the activity of SOD, POD, APX and GR also increased in tomato seedlings [44]. POD activity was also enhanced in cowpea leaves after 1 day of salinity stress [45]. Fundamentally, the high activation of antioxidant enzymes assists plant to be highly tolerant or resistant to stressful conditions [44]. Additionally, the expression of gene encoding antioxidant enzymes increases proportionally to the level of oxidative stress which produces an excessive amount of reactive oxygen species [46]. Under submergence, the concentration of hydrogen peroxide, singlet oxygen, hydroxyl radicals, and superoxide in cells can dramatically increase [12,13], as chemical signals activating the expression of antioxidant enzyme genes in plant such as rice [47]. The gene expression level of antioxidant enzymes in this study was slightly elevated after rice being pre-soaked in PA and VA. 

In previous reports, exogenous application of paclobutrazol (PB), a biosynthesis inhibitor of the phytohormone gibberellins (GA), was reported to promote submergence tolerance in rice [48,49,50]. The pretreatment 24 h of paclobutrazol before flooding showed significant effect on antioxidant enzymes, leaf water potential, and chlorophyll content in sweet potato [51]. However, this was the first to treat exogenous phenolic acids on rice seedlings to promote submergence tolerance, which effectively enhanced the submergence tolerance in rice. Regarding to the submergence tolerance index, the application PA and VA promoted significantly shoot elongation (Table 1). Similarly, Bailey-Serres and Voesenek [52] and Bailey-Serres et al. [53] noted that deep-water rice and most lowland rice genotypes generally adopted the submergence tolerance, characterized by the ethylene-mediated rapid elongation growth promoted by gibberellins, associated with carbohydrate consumption. In deep-water rice, the submergence tolerance was regulated by two ethylene-responsive factors (*ERFs*), *SNORKEL1* (*SK1*), and *SNORKEL2* (*SK2*), that triggered considerable internode elongation via GA during flooding [6,31]. Anandan et al. [54] highlighted that shoot elongation was an important factor adopted by rice to resume aerobic metabolism and to improve carbon fixation in submergence. 

Although Setter and Leureless [48] and then Xiang et al. [50] described that PB showed a negative correlation with shoot elongation of rice, the submergence tolerance of rice was enhanced by increasing the chlorophyll contents, as also observed in this study (Figure 1). It was explained that the response to flooding by enhanced elongation ability when floodwater increases slowly resulting in a partial submergence, as it often does in deep-water and floating rice ecosystems [55]. The elongation ability reduced with consequently greater submergence tolerance when floodwater increases rapidly resulting in complete submergence, as often occurs in flash-flooding [48]. Application of PB also promoted alcohol dehydrogenase activity, and decelerated the consumption of non-structure carbohydrates, droving the expression of several submergence-related genes, such as *CIPK15*, *MPK3*, *SD-1*, and *OsCyt-inv1*, to active the corresponding pathway to enhance submergence tolerance [50]. In this study, antioxidant enzymes including SOD, CAT, APX, POD, and GR were elevated, thus the activation of submergence-related genes, which potentially differed from that of PB and associated to the shoot elongation of rice, should be investigated. 

Although PA and VA showed effective on the stimulation of rice growth in submergence, similar examination should be conducted on other phenolic acids which were detected in anaerobic flooding tolerant rice [24], such as gallic acid, catechol, chlorogenic acid, caffeic acid, syringic acid, vanillin, *p*-coumaric acid, benzoic acid, ellagic acid, and cinnamic acid, to understand the potential of using these compounds for enhancing the submergence tolerance in rice. As observed in this study, mixture of these compounds may provide higher promotion on rice growth in submergence than the application of individual phenolic acids, apparently needs elaboration.

## 4. Materials and Methods

### 4.1. Plant Material and Treatments

Seeds of a rice cultivar namely Koshihikari (*Oryza sativa* L.) harvested in affiliated fields of Hiroshima University, Japan, in 2016, were used. One hundred gram rice seeds were sterilized by soaking in 0.1% NaOCl for 30 min, and then cleaned four times in water. The seeds were then immersed in distilled water again at 30 °C for 24 h. After that, the seeds were germinated at 30 °C in a growth chamber without light. Germinated seeds were separately placed into seedling trays filled with nutrient soil, and grown in a net house for 3 weeks. Subsequently, roots of rice seedlings were soaked in different dilution of 1, 0.1 and 0.01 mM solutions of PA, VA and their corresponding mixtures for 24 h. All treated seedlings were transferred to a water tank (100 cm × 200 cm × 80 cm of width × length × height) for submergence treatment. The seedlings were placed at 20 cm × 10 cm density (total 90 rice seedling per tank), conducted in thrice and repeat twice. After each 3 and six days of submergence, leaves and roots were collected, washed with tap water and then stored at −20 °C for further analysis. The shoot elongation (cm) was measured after de-submerged for 10 days (Table 1). The survival percentages and scores were evaluated according a method described in the International Rice Research Institute (IRRI) [56]. The survival percentage was determined as the plant numbers excluding from death rice seedlings during submergence treatment against total experimented plant numbers. Of which, the scale for scoring submergence included 1: survival 100%; 3: survival 95–99%; 5: survival 75–94%; 7: survival 50–75%; and 9: survival 0–49% (Table A1).

### 4.2. Lipid Peroxidation Measurement

Lipid Peroxidation (LP) was estimated via the MDA content [57] with some minor modifications. An amount of 0.4 g frozen leaves was ground with 2 mL of 0.5% trichloroacetic acid (TCA) using a pestle and mortar, and the mixture was centrifuged for 10 min at 10,000 rpm. The supernatant was transferred to a new tube, and a volume of 20% TCA including thiobarbituric acid was added in the tube which were later incubated at 95 °C for a half-hour and immediately placed in a beaker containing ice. The reaction solution was spectrophotometrically (TU-1800_PC_ UV-vis spectrophotometer, Shimadzu, Co., Ltd., Tokyo, Japan) recorded at 532 nm, and the absorbance values were used for calculation the MDA content based on an extinction coefficient (155 mM^−1^ cm^−1^).

### 4.3. Measurement of Chlorophyll Contents

Chlorophyll contents were quantified according to the method described by Quan et al. [58]. An amount 500 mg of leaves was ground in 2 mL of 85% acetone using a mortar and pestle. The mixture was then centrifuged at 5000 rpm for 5 min. A volume of the above layer (0.5 mL) was transferred to a new tube and diluted with acetone solution for measuring the absorbance at two wavelengths (644 and 663 nm). Contents of chlorophylls a and b (mg/g fresh weight (FW)) were estimated according to the following formulas:

Chlorophyll *a* = 10.3 × Abs_663_ − 0.918 × Abs_644_ (1)

Chlorophyll *b* = 19.7 × Abs_644_ − 3.87 × Abs_663_ (2)

### 4.4. Estimation of Total Phenolic and Flavonoid Contents

Total contents of phenolics and flavonoids were spectrophotometrically estimated followed the methods of Khang et al. [24]. Total phenolic and flavonoid contents were shown as mg gallic acid equivalents (GAE) per gram dry weight (DW) and mg of rutin equivalents (RE) per gram DW, respectively.

### 4.5. Identification and Quantification of Protocatechuic Acid and Vanillic Acid

The column C18 (5 µm × 0.46 cm × 25 cm) connected to the HPLC (JASCO, Tokyo, Japan) system with a LC-Net II/ADC controller, a PU-2089 pump and an UV-2075 detector was used for quantifying the concentrations of PA and VA. The leaf and root extracts in methanol (1 mg/mL) were filtered, and a volume of 5 µL was injected. A gradient elution of absolute methanol (A) and 0.1% acid acetic (B) was run at 1 mL/min speed with a linear increase of A from 5–10% for 5 min, then 10–90% for 45 min, and 10 min cleaning step with only solvent A. The phenolic standard curves were established from different concentrations from 0 to 100 µg/mL for quantification of identified phenolics in the samples.

### 4.6. Enzyme Extraction and Assays

An amount of 0.1 g leaves was homogenized with 1800 µL of PB (pH 6.8) and 200 µL of 1 mM ethylenediaminetetraacetic acid (EDTA). The mixture was centrifuged for 20 min at 15,000 rpm, and the upper portion was kept at −20 °C for further measurement. APX activity was evaluated using the method described by Nakano and Asada [59] with several changes. A volume of 0.6 mL of 0.05 M PB (pH 7.0), 0.1 mL of 0.001 M EDTA, 0.1 mL of 0.005 M ascorbate, 100 μL of enzyme extracts, and 0.1 mL 1 mM H_2_O_2_ were added in a reaction tube, and then the reaction mixture was immediately measured at 29 nm for three minutes. The APX activity was calculated using the extinction coefficient (2.8 mM^−1^ cm^−1^) and expressed as unit/min/g FW. CAT activity was estimated by measuring the decrease of hydrogen peroxide [16]. The reaction included 1.5 mL 0.1 M PB (pH 7.0), 950 μL distilled water, 50 μL enzyme extracts, and 0.5 mL of 75 mM H_2_O_2_. The reduction of H_2_O_2_ was recorded at 240 nm for 3 min, and the CAT activity was calculated using the extinction coefficient (6.93 × 10^−3^ mM^−1^ cm^−1^) and expressed as unit/min/g FW. Activity of SOD was assayed based on the reaction of riboflavin and nitro blue tetrazolium (NBT) [60]. The reaction included 1.5 mL of 0.05 M PB (pH 7.8), 1 mL distilled water, 100 μL of 3 mM EDTA, 100 μL of 200 mM L-methionine, 10 μL of 2.25 mM NBT, 50 μL enzyme extracts, and 100 μL riboflavin in a test tube. After shaking, the reaction was initiated by placing under a lamp (15 W) for 15 min. The control was the mixture without the enzyme extract, and the blank containing the same ingredients with those of the control was not placed under the lamp. The SOD activity was calculated by the subtraction of the control to the sample, and expressed as unit/g FW. POD activity was measured by guaiacol method with some changes [61]. The reaction mixture consisted of 150 μL of 4% guaiacol, 150 μL of 1% H_2_O_2_, 2.66 mL of phosphate buffer (pH 7.0), and 40 μL of enzyme extracts. The absorption was recorded at 465 nm for 180 s. The POD activity was calculated based on the extinction coefficient (25 mM^−1^ cm^−1^) and shown as unit/min/g FW. GR activity was estimated based on the modified method of Foyer and Halliwell [62]. The reaction contained 0.6 mL of 50 mM PB (pH 7.6), 0.1 mL of EDTA (0.003 M), 0.1 mL of 100 μM dihydronicotinamide-adenine dinucleotide phosphate (NADPH), 0.1 mL of 0.001 mM glutathione disulfide (GSSG), and 0.1 mL of enzyme extract. The reaction absorbance was read at 340 nm for 180 s. The activity was calculated using the extinction coefficient (6.2 mM^−1^ cm^−1^) for GSSG and shown as unit/min/g FW.

### 4.7. Gene Expression of Antioxidant Enzymes Using Quantitative Real-Time PCR

The total RNA in leaves was isolated using the RN-Sure Plant Kit (Apro Science, Tokushima, Japan) for examining the gene expression of antioxidant enzyme genes, following manufacturer’s instructions. An amount of 1 μg total RNA was used for synthesizing cDNA using the SuperScript III First-Strand Synthesis System for RT-PCR (Invitrogen, Carlsbad, CA, USA). After diluted to 100 ng, a volume of 2 μL cDNA of each treatment was added to a real-time PCR plate containing 6 μL KAPA SYBR FASTqPCR MasterMix (2X) Universal and primers (1 μL of each forward and reverse primer (10 μM)). The PCR plate was covered by a transparent film and run in the StepOne real-time PCR system (Applied Biosystem Corp., Foster City, CA, USA). The program was set for enzyme activation at 95 °C for 30 s, followed by 40 cycles of denaturing at 95 °C for 5 s and annealing at 60 °C for 60 s. The list of primers is shown in Table 3.

### 4.8. Statistical Analyses

All data were statistically analyzed using one-way analysis of variance (ANOVA), and Tukey’s test was used to compared means using the Minitab 16.0 (Minitab Inc.; State College, PA, USA). Comparisons with *p* < 0.05 were considered significantly different. Pearson’s correlation coefficients (r) among parameters were calculated using SPSS software 18.0 (Chicago, IL, USA). The mRNA increase was estimated using the ∆CT (delta threshold cycle) method [63].

## 5. Conclusions

Enhancement of the antioxidant enzyme system during submergence using chemical treatment is an innovative approach. The use of PA and VA and their mixture was promising for improving rice growth in submergence. This study helped to clarify the effects of exogenous PA and VA on both enzymatic and non-enzymatic antioxidant systems of rice during submergence. Application of mixture of PA and VA were effective and economically efficacious to enhance the submergence tolerance. However, further trials should be performed to examine how PA and VA respond to different water depths, which may help to understand the actual role of the two compounds in strengthening submergence tolerance in rice. 

## Figures and Tables

**Table 1:** Effects of exogenous application of phenolic acids on shoot height of rice in submergence.

Treatments	Shoot Height(cm)	Survival Percentage	Scale
Control	5.19 ± 0.08 ^c^ (0.0)	75.56 ± 6.46 ^d^	5
PA 0.01	4.56 ± 0.38 ^c^ (12.1)	83.33 ± 4.35 ^c^	5
PA 0.10	5.63 ± 0.38 ^b^ (−8.48)	95.56 ± 3.51 ^b^	3
PA 1.00	6.27 ± 0.28 ^ab^ (−20.8)	100.00 ± 0.00 ^a^	1
VA 0.01	4.45 ± 0.36 ^c^ (14.26)	85.19 ± 5.67 ^c^	5
VA 0.10	5.46 ± 0.17 ^b^ (−5.20)	96.30 ± 2.65 ^b^	3
VA 1.00	6.37 ± 1.41 ^ab^ (−22.4)	96.67 ± 1.33 ^b^	3
M 0.01	6.01 ± 0.61 ^ab^ (−15.7)	97.78 ± 0.23 ^b^	3
M 0.10	6.16 ± 1.03 ^ab^ (−18.69)	100.00 ± 0.00 ^a^	1
M 1.00	6.33 ± 2.96 ^a^ (−21.97)	100.00 ± 0.00 ^a^	1

PA: Protocatechuic acid; VA: vanillic acid; M: Mixture of PA and VA; 1.0, 0.1, and 0.01 are different dilution at 1.0, 0.1, and 0.01 mM; Scale 1: 100%, 3: 95–99%, 5: 75–94% survival (Table A1); Different letters in same column indicate significantly difference at *p* < 0.05; Values in columns are means ± SD (standard deviation); Values in the parentheses are inhibition over control (%). Minus values in parentheses are promotion over control (%).

**Table 2:** Correlation (*R^2^*) among measured parameters relating to phenolics, flavonoids, lipid peroxidation, chlorophylls and antioxidant enzyme activities.

	Phenolics	Flavonoids	MDA	Chla	Chlb	CAT	SOD	APX	POD	GR
Phenolics	1									
Flavonoids	0.579 *	1								
MDA	−0.158	0.309	1							
Chla	0.579 *	0.640 *	0.030	1						
Chlb	0.521	0.797 **	0.364	0.832 **	1					
CAT	0.228	0.216	−0.565	−0.120	−0.187	1				
SOD	0.818 **	0.793 **	−0.188	0.644 *	0.566	0.413	1			
APX	0.865 **	0.291	−0.542	0.322	0.178	0.484	0.721 **	1		
POD	−0.090	−0.219	0.680 *	−0.100	0.044	−0.773 **	−0.398	−0.274	1	
GR	0.364	0.208	−0.630 *	0.336	0.135	0.470	0.564	0.510	−0.702 *	1

*, **: correlation is significant at the 0.05 and 0.01 level, respectively; CAT: Catalase; GR: Glutathione reductase; SOD: Superoxidase; POD: Peroxidase; APX: Ascorbate peroxidase; MDA: Malondialdehyde; Chla: Chlorophyll *a*; Chlb: Chlorophyll *b*.

**Table 3:** Primer sequences of the selected antioxidant enzyme genes.

Genes	Primer Sequences
*SOD*	Forward: GGCTTGCATACAAACCTGAA Reverse: CTGACTGCTTCCCATGACACCAT
*CAT*	Forward: GTCGATTGGTGTTGAACAGG Reverse: AGGACGACAAGGATCAAACC
*APX*	Forward: GACTCTTGGAGCCCATTAGG Reverse: AGGGTGAAAGGGAACATCAG
*POD*	Forward: TTAGGGAGCAGTTTCCCACT Reverse: AGGGTGAAAGGGAACATCAG
*GR*	Forward: TTGGTGGAACGTGTGTTCTT Reverse: TCTCATTCACTTCCCATCCA
*Actin*	Forward: TGGTCGGAATGGGACAGAAG Reverse: CTCAGTCAGGAGAACAGGGT

## Appendix A

**Table A1:** Scale for scoring submergence tolerance of rice.

Scale	Survival (%)
1	100
3	95–99
5	75–94
7	50–75
9	0–49

The rice seedlings were de-submerged for 10 days, and then recorded the plant height and scored according to IRRI’s method (IRRI, 2002).
